# X26nt-mediated recruitment of eIF4A2 facilitates CCND1 translation to drive endothelial cell cycle progression

**DOI:** 10.1016/j.gendis.2025.101667

**Published:** 2025-05-02

**Authors:** Xinghe Zhao, Xiaocui Chen, Zheyi Chen, Lisong Shen, Lingfang Zeng, Junyao Yang

**Affiliations:** a Department of Laboratory Medicine, Xin Hua Hospital, Shanghai Jiao Tong University School of Medicine, Shanghai 200092, China; b Faculty of Medical Laboratory Science, College of Health Science and Technology, Shanghai Jiao Tong University School of Medicine, Shanghai 200092, China; c Institute of Artificial Intelligence Medicine, Shanghai Academy of Experimental Medicine, Shanghai 200092, China; d Qingdao Municipal Hospital, Qingdao 266011, China; e Institute of Artificial Inte School of Cardiovascular Medicine and Sciences, King's College London British Heart Foundation Centre of Excellence, Faculty of Life Science and Medicine, King's College London, London SE5 9NU, United Kingdom

Dysregulation of cell cycle control plays a pivotal role in tumor progression. Acting as a crucial regulator of the G1/S phase transition, dysregulation of cyclin D1 (*CCND1*) can disrupt the delicate balance between cell proliferation and quiescence, leading to uncontrolled cell cycle progression.^1^ Endoplasmic reticulum stress influences the cell cycle through myriad pathways. During endoplasmic reticulum stress, the inositol-requiring enzyme 1 alpha (IRE1α) can be activated, and its endoribonuclease domain cleaves unspliced X-box binding protein 1 (*XBP1u*) mRNA, generating a spliced XBP1 (*XBP1s*) and a non-coding RNA, whose length is 26 nt (named exosomal 26-nt-long ncRNA, *X2*6nt).^2^^,^^3^ Studies have demonstrated that *XBP1s* can induce the expression of pro-angiogenic factors, promoting the formation of new blood vessels.^4^^,^^5^ Our previous study demonstrated that gastric cancer exosome-derived *X2*6nt induces tumor-associated angiogenesis via promoting endothelial cell migration targeting vascular endothelial-cadherin.^2^ However, the exact mechanisms by which *XBP1* splicing regulates endothelial cell proliferation are still not fully understood.

When synchronizing human umbilical vein endothelial cells (HUVECs) at the late G1 phase by double thymidine block and then releasing it to re-enter the cell cycle, we observed an increase in the number of cells in the G2/M phase at 6 h and 9 h after release (Fig. S1A). We collected samples at the same indicated time points and performed PCR and Western blot analysis. The results showed that the expression level of the spliced form of XBP1 (*XBP1s*) was consistently increased at 6 h and 15 h (Fig. 1A, B; Fig. S1B). Vascular endothelial growth factor (VEGF) induced endoplasmic reticulum stress resulting in up-regulation of the cell cycle-related protein CCND1. Upon *XBP1* knockdown or inhibition of *XBP1* splicing (*IRE1α* knockdown), VEGF-induced up-regulation of CCND1 was eliminated (Fig. S2). Based on this, we hypothesized that *XBP1* splicing could play a crucial role in mediating cell cycle in HUVECs. However, further experiments revealed that over-expression of *XBP1s* (Ad-XBP1s) suppressed the expression of CCND1 and other cell cycle-related proteins in HUVECs (Fig. 1C, D; Fig. S3). These findings indicated that *XBP1s* may not be the key factor regulating cell cycle-related protein CCND1. Thus, we focused on *X2*6nt and hypothesized that it could act as a mediator of the cell cycle. Knockdown of *XBP1* significantly reduced cell proliferation, and its *X2*6nt, not *XBP1s*, could restore the proliferation capability (Fig. S4).

**Figure 1:**
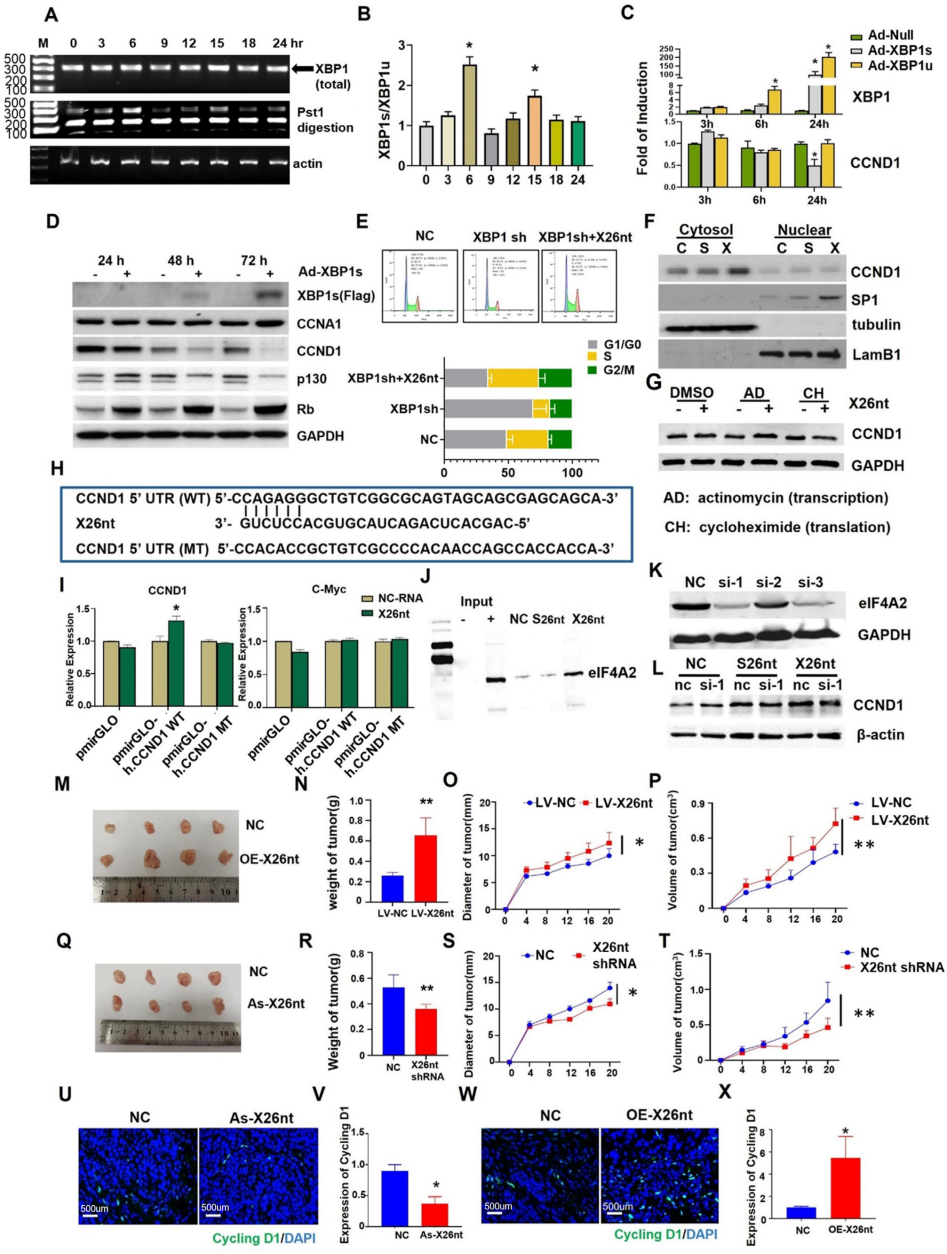
*X2*6 nt can increase *CCND1* through binding to the 5′ UTR of *CCND1* mRNA mediated by EIF4A2, facilitate the proliferation of HUVECs, promote tumor angiogenesis, and therefore contribute to tumor progression. **(A)** HUVECs were synchronized at the late G1 phase by double thymidine block, and then released to re-enter the cell cycle. Cells were harvested at the time indicated after release. PCR analysis of *XBP1* (total) and *XBP1s* expression after release at different times (*n* = 3). **(B)** Quantitative analysis *XBP1s/XBP1u* rate of (A). **(C)** HUVECs were infected with Ad-*XBP1s* and Ad-*XBP1u*, and *XBP1* and *CCND1* expression were analyzed by PCR. Ad-null was introduced as a control. **(D)** Western blots of XBP1s, CCNA1, CCND1, P130, RB, and E2F2 of Ad-*XBP1s*-infected-HUVECs were collected at 24 h, 48 h, and 72 h. GAPDH was introduced as the loading control. **(E)** Flow cytometry analysis showed the cell cycle distribution of HUVECs transfected with negative control (NC) or *XBP1* shRNA, and cells transfected with *XBP1* shRNA cocultured with *X2*6nt. Bar graphs show the percentages of HUVECs in the G0/G1, S, and G2/M phases. **(F)** Western blot analysis of cytosol and nuclear proteins collected from HUVECs cocultured with negative control, *S2*6nt, or *X2*6nt. TUBULIN and LAMB1 were introduced as the loading controls. **(G)** The protein expression of CCND1 with the treatment of actinomycin (AD) and cycloheximide (CH) in HUVECs cocultured with or without *X2*6nt. **(H)** The schematic showing potential *X2*6nt binding sites in the *CCND1* mRNA 5′ UTR. **(I)** The luciferase assay of HUVECs co-transfected firefly luciferase reporter plasmid containing either wild-type (pmirGLO-h. CCND1 WT) or mutant *CCND1* mRNA 5′ UTR with pmirGLO-h. *CCND1* mRNA cocultured with *X2*6nt or *NC-RNA*. ∗*p* < 0.05. **(J)** Western blots of EIF4A2 in cells cocultured with *NC*, *S2*6nt, and *X2*6nt. **(K)** Immunoblots of EIF4A2 in HUVECs transfected with three different *si-EIF4A2* plasmids. The scrambled *EIF4A2* inhibitors were transfected as the control (nc). **(L)** Western blot analysis of CCND1 in nc and si-1 HUVECs co-cultured with *S2*6nt and *X2*6nt. β-ACTIN was introduced as the loading control. **(M**–**P)** The formed tumors from BGC-823 cells transfected with X26nt-overexpressing (*OE-X2*6nt) lentivirus and control lentivirus (Control) were isolated and compared. Analysis of tumor diameter, volume, and weight in each group is shown. **(Q**–**T)** The BGC-823 cells were subcutaneously injected into the *BALB/c* nude mice to create a tumor-implanted model. When the tumor appeared, X26nt antisense RNA (*As-X2*6nt) plasmid and mock control (NC) were injected into the tumor every other day before the mice were sacrificed. Analysis of tumor diameter volume and weight in each group is shown. **(U**–**X)** Immunofluorescence staining of CCND1/DAPI in tumors treated with *X2*6nt asRNA plasmid and NC, as well as X26nt OE lentivirus and control. ∗*p* < 0.05 and ∗∗*p* < 0.01 versus the control. Columns, mean (B–G, I–L: *n* = 3; M–X, *n* = 4); bars, standard deviations. The data presented are representative or average of three independent experiments. X26nt, exosomal 26-nt-long ncRNA; CCND1, cyclin D1; EIF4A2, eukaryotic translation initiation factor 4A2; XBP1, X-box binding protein 1; XBP1s, spliced X-box binding protein 1; CCNA1, cyclin A1; E2F2, E2F transcription factor 2; LAMB1, laminin subunit beta 1.

Our former studies showed that *X2*6nt could promote the proliferation, migration, and tube formation of endothelial cells.^3^ To investigate the role of *X2*6nt in the proliferation of HUVECs, we conducted loss-of-function assays by adding *X2*6nt to *XBP1s* knockdown cells and observed that *X2*6nt significantly reversed the cell cycle arrest in the G1/G0 phase induced by *XBP1s* knockdown cells (Fig. 1E; Fig. S5A). We also constructed a mutant *S2*6nt as a negative control for *X2*6nt and examined the protein levels of CCND1 and specificity protein 1 (SP1) in cytoplasmic and nuclear fractions within *S2*6nt or *X2*6nt overexpression (Fig. 1F). The findings confirmed that *X2*6nt genuinely enhanced the cytoplasmic expression of CCND1. The expression of X26nt was down-regulated in Ad-XBP1s, which indicated that X26nt was the effective factor in promoting the expression of CCND1 and cell cycle (Fig. S5B). To explore the mechanism underlying the promotion of *CCND1* by *X2*6nt, we treated HUVECs with actinomycin D and cycloheximide. Actinomycin D can inhibit RNA formation and therefore reflect the efficiency of transcription. Cycloheximide can modulate translation efficiency by inhibiting protein production. The results demonstrated that *X2*6nt could still enhance the expression of CCND1 after RNA transcription was inhibited, but its effect was lost when protein translation was inhibited (Fig. 1G; Fig. S6A). This suggests that *X2*6nt may regulate the translation efficiency of *CCND1*.

We conducted further investigations to understand the mechanism underlying *X2*6nt interacting with *CCND1*. Using bioinformatics tools, we predicted the binding site of *X2*6nt on 5′ UTR of *CCND1* mRNA (Fig. 1H). Subsequently, a dual-luciferase reporter assay demonstrated that *X2*6nt significantly enhanced the activity of firefly luciferase reporter containing the wild-type 5′ UTR of *CCND1* mRNA. Furthermore, this effect was abolished when the predicted binding site in 5′ UTR was mutated. Interestingly, the expression level of *C-MYC* was not affected by either *X2*6nt or mutated 5′ UTR of *CCND1* mRNA (Fig. 1I). To further validate the direct protein target of *X2*6nt in proliferating endothelial cells, we synthesized biotinylated X26nt (Bio-X26nt), incubated it with VEGF-treated endothelial cells, and performed affinity purification using streptavidin beads. Mass spectrometry analysis demonstrated that eukaryotic translation initiation factor 4A2 (EIF4A2) exhibited a higher score in the VEGF-treated group, indicating its potential involvement in *X2*6nt-mediated effects. Importantly, EIF4A2 is known to play a critical role in protein translation. This observation was further substantiated by Western blot analysis, which displayed the formation of a complex involving X26nt and EIF4A2 (Fig. 1J; Fig. S7). To substantiate that X26nt could trigger CCND1 via its interaction with EIF4A2, we utilized siRNA to suppress EIF4A2 (Fig. 1K). The findings revealed that the capacity of X26nt to enhance CCND1 expression was nullified when EIF4A2 was suppressed, corroborating our initial hypothesis (Fig. 1L; Fig. S6B).

Our former research also found that X26nt was significantly higher in gastric cancer serum and tissues, and X26nt accelerated tumor angiogenesis.^3^ Furthermore, we found that the levels of X26nt were remarkably higher in gastric cancer cells than in HUVECs (Fig. S8). To ascertain the role of *X2*6nt in promoting tumor growth, a tumor-implanted model was constructed by subcutaneously injecting loss-of-function (via shRNA lentivirus-mediated knockdown of *X2*6nt) and gain-of-function (via adenoviral transfer of *X2*6nt) BGC-823 cells (human gastric adenocarcinoma cell line) into the back of the armpit of nude mice. Analysis of changes in tumor weight and volume trends revealed that *X2*6nt overexpression promoted tumor growth (Fig. 1M−P), while its knockdown had the opposite effect (Fig. 1Q–T). Additionally, we noted that *X2*6nt knockdown resulted in a decrease in CCND1, while inversely, *X2*6nt overexpression led to an increase in CCND1 (Fig. 1U–X). Consequently, we concluded that *X2*6nt could increase *CCND1* expression and promote tumor growth *in vivo*.

Our former study also revealed that *X2*6nt could inhibit the expression of vascular endothelial-cadherin and enhance the migration and tube formation abilities of endothelial cells.^3^ Our subsequent results confirmed that loss of *X2*6nt induced cell cycle arrest in the G1/G0 phase. Our discovery showed that *X2*6nt could enhance the translation efficiency of *CCND1* by binding to the 5′ UTR of *CCND1* mRNA, with the involvement of EIF4A2. This could promote the cell cycle progression of HUVECs in the late G1/S phase. *In vivo* experiments further confirmed that *X2*6nt accelerated the expression of *CCND1* and tumor growth. As far as we are concerned, there is no evidence showing that the increasing level of *X2*6nt has a direct effect on the cell cycle. In our prior research, we did observe that the levels of *X2*6nt were significantly elevated in the serum from gastric cancer patients compared with normal serum. Hence, we propose that the overexpressed *X2*6nt might be secreted via gastric cancer cell-derived exosomes and subsequently internalized by HUVECs.^3^ This could lead to the proliferation and migration of endothelial cells, thereby encouraging tumor angiogenesis (Fig. S9). Ultimately, *X2*6nt may be a weapon for tumor cells to modify the tumor microenvironment, and it may serve as a potential target for tumor therapy.

## CRediT authorship contribution statement

**Xinghe Zhao:** Visualization, Investigation. **Xiaocui Chen:** Methodology, Investigation, Formal analysis, Data curation. **Zheyi Chen:** Methodology, Investigation, Formal analysis, Data curation. **Lisong Shen:** Writing – review & editing, Writing – original draft, Resources, Funding acquisition, Formal analysis, Conceptualization. **Lingfang Zeng:** Writing – review & editing, Formal analysis, Data curation, Conceptualization. **Junyao Yang:** Writing – review & editing, Writing – original draft, Investigation, Funding acquisition, Formal analysis, Conceptualization.

## Ethics declaration

The animal study protocol was approved by the Institutional Review Board of the Laboratory Animal Ethical and Welfare Committee Xinhua Hospital Affiliated to Shanghai Jiao Tong University School of Medicine (approval number: XHEC-F-2024-002, date of approval: 2024-01-30).

## Funding

This research was funded by the 10.13039/501100001809National Natural Science Foundation of China (No. 81802082 to Junyao Yang; 81672363 to Lisong Shen), the Natural Science Foundation of Shanghai Science and Technology Innovation Action Plan (China) (No. 21ZR1441500 to Junyao Yang), and the Shanghai "Rising Stars of Medical Talent" Youth Development Program-Clinical Laboratory Practitioners Program (China) (No. 2019016 to Junyao Yang).

## Conflict of interests

The authors declared no conflict of interests. The funders had no role in the design of the study; in the collection, analyses, or interpretation of data; in the writing of the manuscript; or in the decision to publish the results.
