# Supplementary material for: X26nt-mediated recruitment of eIF4A2 facilitates CCND1 translation to drive endothelial cell cycle progression
Source: Genes Dis. 2025 May 2;12(6):101667. doi: 10.1016/j.gendis.2025.101667 (PMC12281298; doi:10.1016/j.gendis.2025.101667)
Supplement: Multimedia component 1 [file mmc1.docx]

**Materials and Methods**

**Materials**

All cell culture serum and media were purchased from Gibico, while cell culture supplements were purchased from Sigma. The antibodies against Cyclin D1 (1:500; ab40754), CCNA1 (1:1000; ab270940), p130 (1:1000; ab76234), eIF4A2 (1μg/ml; ab31218), Rb (1:2000; ab181616), SP1 (1:1000; ab231778), tubulin (1:5000; ab7291), LamB1 (1:1000; ab109293), flag (1:1000; ab205606) and GAPDH (1:2000; ab8245) were from Abcam. The antibody against XBP1 (M-186, sc-7160) was from Santa CruZ Biotech. All secondary antibodies were from Dakocytomation and Cell Signaling Technology. All microRNA reagents were purchased from Thermo Fisher Scientific. DMSO (D2650), DAPI (D9542), actinomycin (SBR00013), cycloheximide (239765) were purchased from Sigma.

**Cell culture**

Human umbilical vein ECs (HUVECs, ATCC-PCS-100-010) were obtained from Scien-Cell Research Laboratories and cultured on 0.04% gelatin-coated flasks in M199 medium supplemented with 1ng/ml β-EC growth factor, 3ug/ml EC Growth Supplement from bovine neural tissue, 10ug/ml heparin, 1.25μg/ml thymidine, 10% FBS, 100ug/ml penicillin and streptomycin. The cells were split every three days at a ratio of 1:5. The GC cell line BGC-823 cell was purchased from the Cell Bank of Type Culture Collection of the Chinese Academy of Sciences and cultured in Dulbecco's Modified Eagle's Medium (DMEM; Gibco, Life Technologies) with 10% FBS and antibiotics (100 µg/mL penicillin and 100 µg/mL streptomycin). The cell culture was placed in humidified air at 37°C with 5% CO2.

**Flow cytometry analysis**

Cells were collected and fixed with 70% ethanol at 4°C for 24 h and were then stained with propidium iodide (PI, Beyotime, C1052). Flow cytometry analysis of the cell cycle was performed using a FACS Calibur (BD Biosciences, San Diego, CA). The data were analyzed using FlowJo (TreeStar, Ashland, OR, USA) and ModFit (BD Bio-science) software.

**Transfection assay**

For adenoviral infection, HUVECs were incubated with Ad-null, or Ad-XBP1u (unspliced XBP1) or Ad-XBP1s (spliced XBP1) virus at 10 multiplicity of infection (MOI) for 6h, and then cultured in fresh complete growth medium for time duration indicated in figure legends. For shRNA lentiviral infection, HUVECs were incubated with 100 transfection unit/cell of non-target shRNA or XBP1 shRNA or IRE1α shRNA lentivirus in the presence of 10mg/mL polybrene for 16h, followed by culture in fresh complete growth medium for 72h and subjected to further treatments. For in vitro transfection of X26nt mimics, X26nt inhibitors (X26nt asRNA), X26nt-overexpressing (X26nt OE) lentivirus, and negative control (Sangon/GeneChem) were transfected using Lipofectamine 3000 (Life Technologies) according to the manufacturer's instructions. Transfection cells were collected and used for further analysis after 24 or 48 hours.

**RNA extraction and quantitative real-time PCR (qRT-PCR)**

The total RNA was isolated using the RNeasy Mini Kit (QIAGEN) following the manufacturer's protocol. The concentration and quality of the total RNA were evaluated using the Nanodrop Spectrophotometer (Thermo Fisher Scientific). Two micrograms of RNA were converted into cDNA using the Improm-II reverse transcription system (Promega). For real-time PCR (qPCR), 20 ng of cDNA was amplified using a real-time PCR SYBR master mix (Applied Biosystems). Primers for qPCR were designed with the Primer Express Software (Applied Biosystems) as follows: XBP1u forward 5′-TGCTGAGTCCGCAGCACTAG-3′, XBP1s forward 5′-TGCTGAGTCCGCAGCAGGTG-3′and a common reverse primer 5′-GCTGGCAGGCTCTGGGGAAG-3′; CCND1 forward 5’-GCTGCGAAGTGGAAACCATC-3’ and reverse 5’-CCTCCTTCTGCACACATTTGAA-3’; c-Myc forward 5’-GTCAAGAGGCGAACACACAAC-3’ and reverse 5’- TTGGAC-GGACAGGATGTATGC-3’; β-actin 5’-CACAACTGGGACGACATGGAG-3’ and 5’-TTCATGAGGTAGTCAGTCTGG-3’ as internal control. The U6 sense primer was 5′-TGGAACGCTTCACGAATTTGCG-3′ and the antisense primer was 5′-GGAACGATACAGAGAAGATTAGC-3′. The X26nt primers are as follows: the outer primer used for primary amplification was 5′-GGTTTTCCCAGTCACGACGCTGCACTCAGACTAC-3′ and 5′-CAGCTATGACCATGATTACGCAGAGGTGCAC-3′; the second-round amplification using inner primer was 5′-GGTTTTCCCAGTCACGACG-3′ and 5′-CAGCTATGACCATGATTACG-3′.

The XBP1 mRNA splicing was detected by RT-PCR followed by Pst Ⅰ digestion. Half of the XBP1 PCR products were subjected Pst I digestion (Promega) for distinguishing the spliced and unspliced XBP1, while the remaining half was used for total XBP1. PCR product and Pst I digests were analysed in 2% agarose gel and images were assessed with BioSpectrum AC Imaging System and VisionWorksLS software. PCR product was 300bp, digestion with Pst I (site sequence highlighted in red) gave 156bp + 144bp fragments. Primers of XBP1 were designed as follows: forward: 5’-AGAAAACTCATGGCCTTGTAG-3’ and reverse: 5’-GaaGGGCATTTGAAGAACATG-3’. For realtime PCR (qPCR), 20ng cDNA was amplified by a real time PCR SYBR master mix (Applied Biosystems).

3% low melting agarose gel was used to separate XBP1u and XBP1s Primer set for XBP1 was forward: 5'-CCTTGTAGTTGAGAACCAGGAG-3’ and reverse: 5'-GGTCCAAGTTGTCCAGAATGC-3’, 252bp was for XBP1u and 226bp was for XBP1s. Primer set for beta-actin was forward: 5'-CACAACTGGGACGACATGGAG-3’ and reverse: 5'-TTCATGAGGTAGTCAGTCTGG-3’.

**Western blot**

Equal amounts of protein extracts were resolved on a 10% sodium dodecyl sulfate-polyacrylamide gel (SDS-PAGE) and subsequently transferred onto a PVDF membrane (IPFL00010; Merck Millipore). The membranes were then incubated with primary antibodies and Anti-rabbit IgG (H + L; DyLight 800 Conjugate; 5151S; Cell Signaling Technology). GAPDH and β-actin were employed as endogenous controls, and densitometry analysis was conducted using ImageJ software (NIH).

**Cell proliferation assay**

Cell proliferation assay was performed in vitro according to the manufacturer's guidelines (C0071S, Beyotime). The cells were photographed using an Olympus CKX53 Imaging System (Olympus Corporation) with an excitation wavelength of 495 nm.

**Luciferase reporter assay**

Wild-type and mutated CCND1 5′UTRs were synthesized and cloned into pmirGLO vector (luciferase report vector, Promega). HUVECs were seeded at 1 × 10^5 cells per well in 24-well plates the day prior to transfection. Cells were transfected with pmirGLO luciferase expression construct containing the 5′UTR of target gene, pRL-TK Renilla luciferase vector (Promega), and mimics or negative control RNA (Ambion). After 48 hours transfection, luciferase activities were measured using the Dual-Luciferase Reporter Assay System (Promega) and normalized to Renilla luciferase activity. All experiments were performed in duplicate with data pooled from three independent experiments

**LC-MS/MS analysis, protein identification and quantification**

The sample was separated using the EASY-nLC 1200 Liquid Chromatography System (Thermo Scientific, USA) and loaded onto a 50 µm × 15 cm Acclaim PepMap RSLC column (Thermo Scientific, USA) at a flow rate of 300 nL/min. The gradient conditions were as follows: 0%-6% buffer B (0.1% FA, 80% acetonitrile) for five minutes, 6%-28% buffer B for 45 minutes, 28%-38% buffer B for 50 minutes, 38%-100% buffer B for 50 minutes, and hold at 100% buffer B for 60 minutes. After separation, the sample was detected using the Q Exactive Plus mass spectrometer (Thermo Scientific, USA). The mass over charge (m/z) range of the precursor ion was set from 350 to 1850 in the MS scan, while MS spectra were obtained at a resolution of 70,000. Raw MS data were subjected to identification using Mascot 2.5. The following parameters were used for protein identification with Mascot 2.5: 2 as the maximum number of missed cleavages, ±20 ppm as the precursor mass tolerance, 0.1 Da as the fragment mass tolerance, iTRAQ-8-plex as the modification groups from the Quan Method, and Oxidation (M), Acetyl (Protein N-term), and Deamidated (NQ) as dynamic modifications. Carbamidomethyl (C) was used as the static modification, decoy was used as the database pattern, and a peptide FDR of ≤0.01 was set. In the quantification analysis, the protein ratios were calculated as the median of only the unique peptides of the protein using Proteome Dis-coverer 2.1. All peptide ratios were normalized by the median protein ratio to ensure that the median protein ratio was 1 after normalization.

**Pre-mir RNAs and siRNA transfection**

For the transfection assay, HUVECs were seeded in triplicate in 6-well plates at a density of 5 × 10^4 cells/well, 24 hours prior to transfection. Prior to transfection, the cells were treated with serum and antibiotics-free M199 medium for 1 hour. The transfection of 20g/mol of pre-miRNAs into HUVECs was carried out using 9μl of lipofectamine RNAi Max (Thermo Fisher Scientific), following the provided procedure. After 6 hours, the transfection solution was removed, and fresh complete growth medium was added. The cells were then incubated and subjected to further treatment as indicated in the figure legends.

For the siRNA transfection assay, HUVECs were seeded in 25ml flasks 24 hours prior to transfection. Similar to the pre-miRNA transfection, the cells were treated with serum and antibiotics-free M199 medium for 1 hour prior to transfection. Transfection of 10μl of 10μmol/L control siRNA or eIF4A2 siRNA (sc-36093) into HUVECs was carried out using 12μl of Lipofectamine RNAi max (Invitrogen), following the provided protocol. Fresh complete growth medium was added 6 hours post-transfection and incubated for 48h.

**In vivo tumor xenografts**

In the in vivo studies, we obtained 4-6-week-old male BALB/c nude mice from the Jiangsu Jicui Yaokang Biotechnology Company. To begin, BGCs that were transfected with X26nt OE lentivirus and control lentivirus were subcutaneously injected into the nude mice. Another group of mice received subcutaneous injections of BGCs, and we administered 15 μg of pCMV-X26nt asRNA and empty pCMV vector control to the tumors every other day once visible tumors appeared. The mice were regularly examined until they were sacrificed. Tumor size was measured using a digital caliper, and tumor volume was calculated using the following formula: volume = 0.5 × width² × length. All animal procedures were conducted with the approval of the Laboratory Animal Ethical and Welfare Committee Xinhua Hospital Affiliated to Shanghai Jiao Tong University School of Medicine (Approval number: XHEC-F-2024-002).

**Immunofluorescence staining**

The cells on slides were fixed with 4% paraformaldehyde at room temperature (RT) for 15min and permeabilised with 0.1% Triton X-100/PBS at RT for 15min, followed by blocking with diluted normal serum (1:20) for 1h, incubation with diluted primary and secondary antibodies at 37ºC for 1h and 45min, respectively. Nucleus was counterstained with DAPI. Images were taken by using SP5 confocal microscope (Leica, Germany) and processed by Adobe Photoshop software.

**Statistical Analysis**

Data expressed as the mean ± S.E. were analyzed using GraphPad Prism software (version 6) with t test for pair-wise comparisons or analysis of variance, when t test was inappropriate, followed by Dunnett's multiple comparison tests, and significance was depicted by asterisks (*, p < 0.05; **, p<0.01).

**Figure S1**


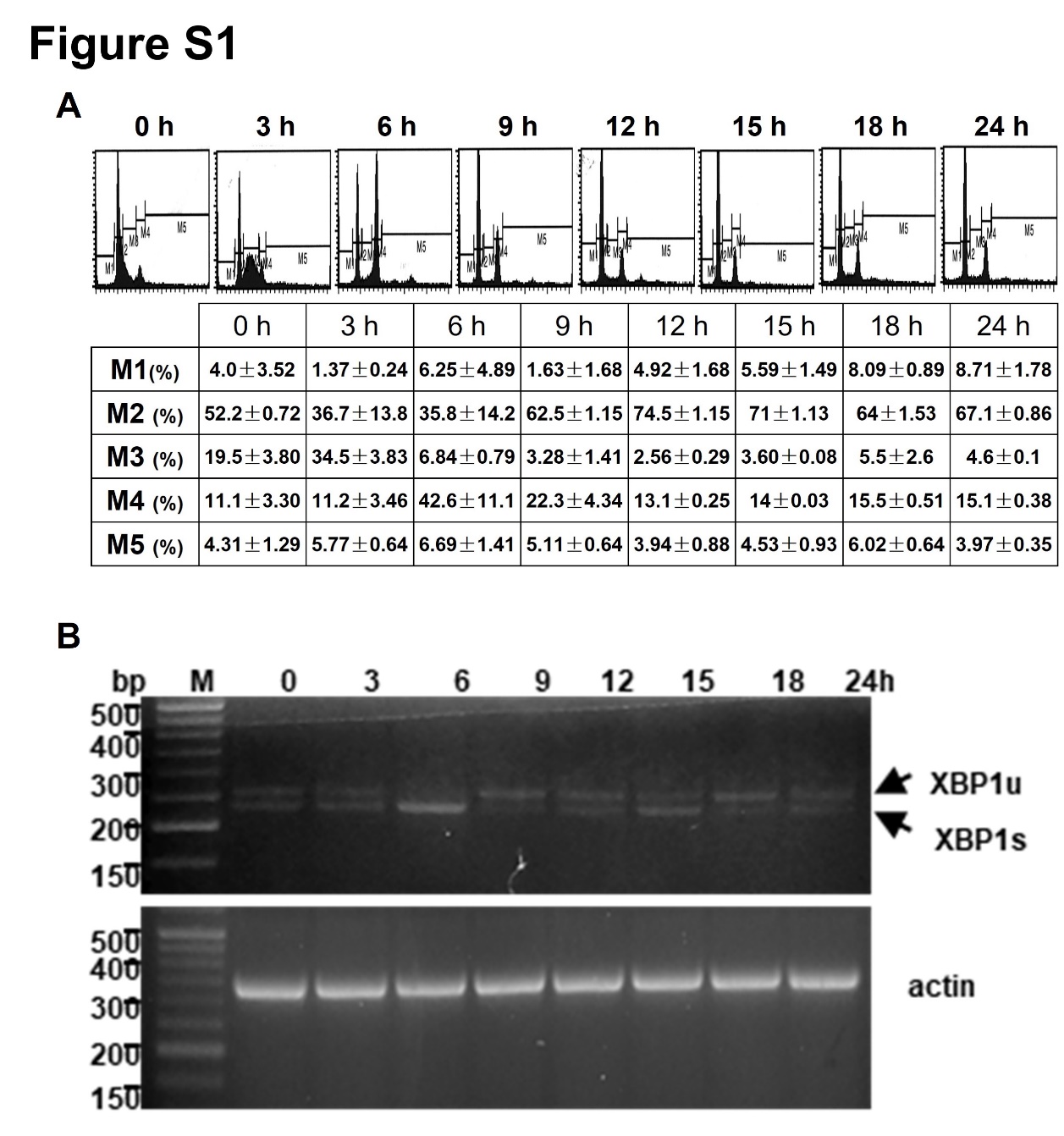


**Figure S1: *XBP1* splicing is a cell cycle-related event.**

HUVECs were synchronized at late G1 phase by double thymidine block, then released to re-enter the cell cycle. Cells were harvested at time indicated after release, followed by cell cycle analysis. Data presented are representative or average of three independent experiments. (B) 3% low melting agarose gel was used to separate *XBP1u* (252bp) and *XBP1s* (226bp).

**Figure S2**


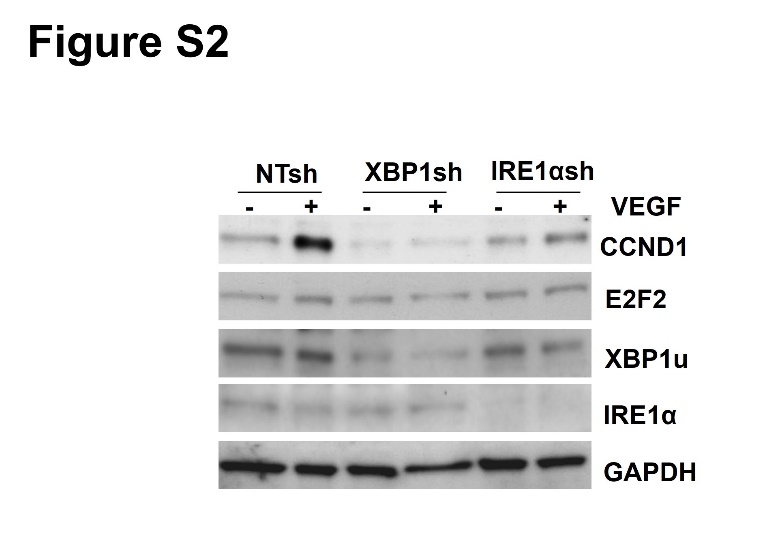


**Figure S2: XBP1 splicing promoted expression of CCND1 mediated by VEGF.**

*XBP1* and *IRE1α* knockdown cells were constructed and treated with VEGF. Western blot analysis showed the expression of CCND1 and E2F2. GAPDH was introduced as loading control (*n = 3*), **P<0.05*.

**Figure S3**


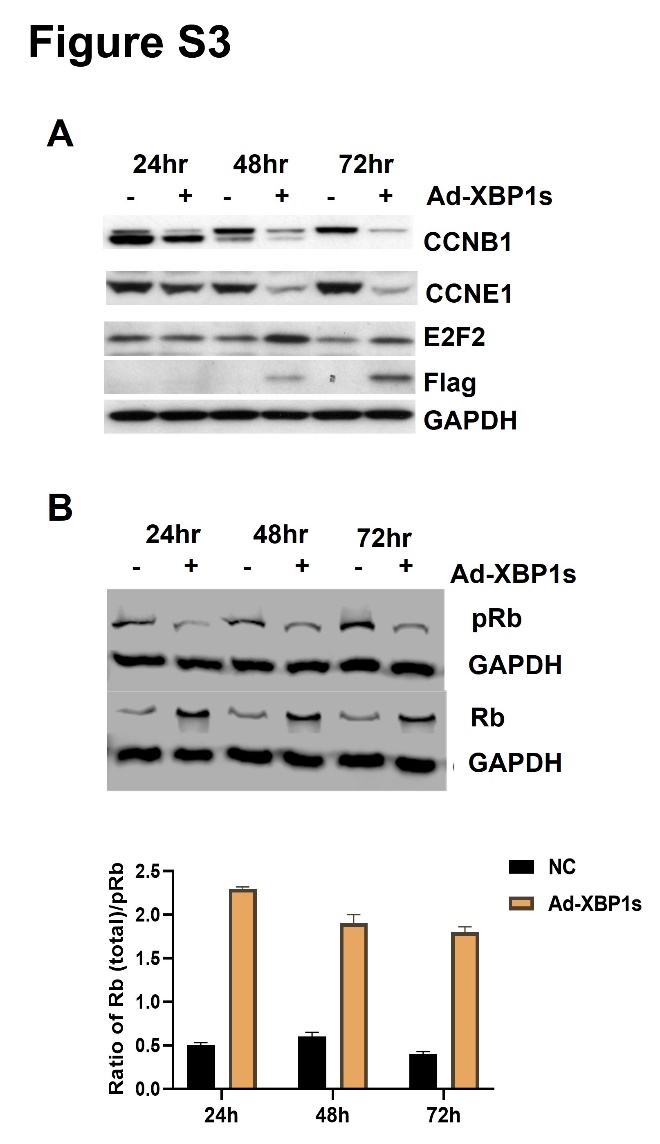


**Figure S3: *XBP1s* showed suppressed effect on some cell cycle-related proteins.**

HUVECs were infected with Ad-XBP1s for 24h, 48h, 72h and collected protein. (A)Western blot of CCNB1, CCNE1, E2F2. GAPDH was introduced as loading control. (B) Western blot of pRb and Rb, as well as analysis of Rb/pRb ratio. GAPDH was introduced as loading control (*n = 3*), **P<0.05*.

**Figure S4**


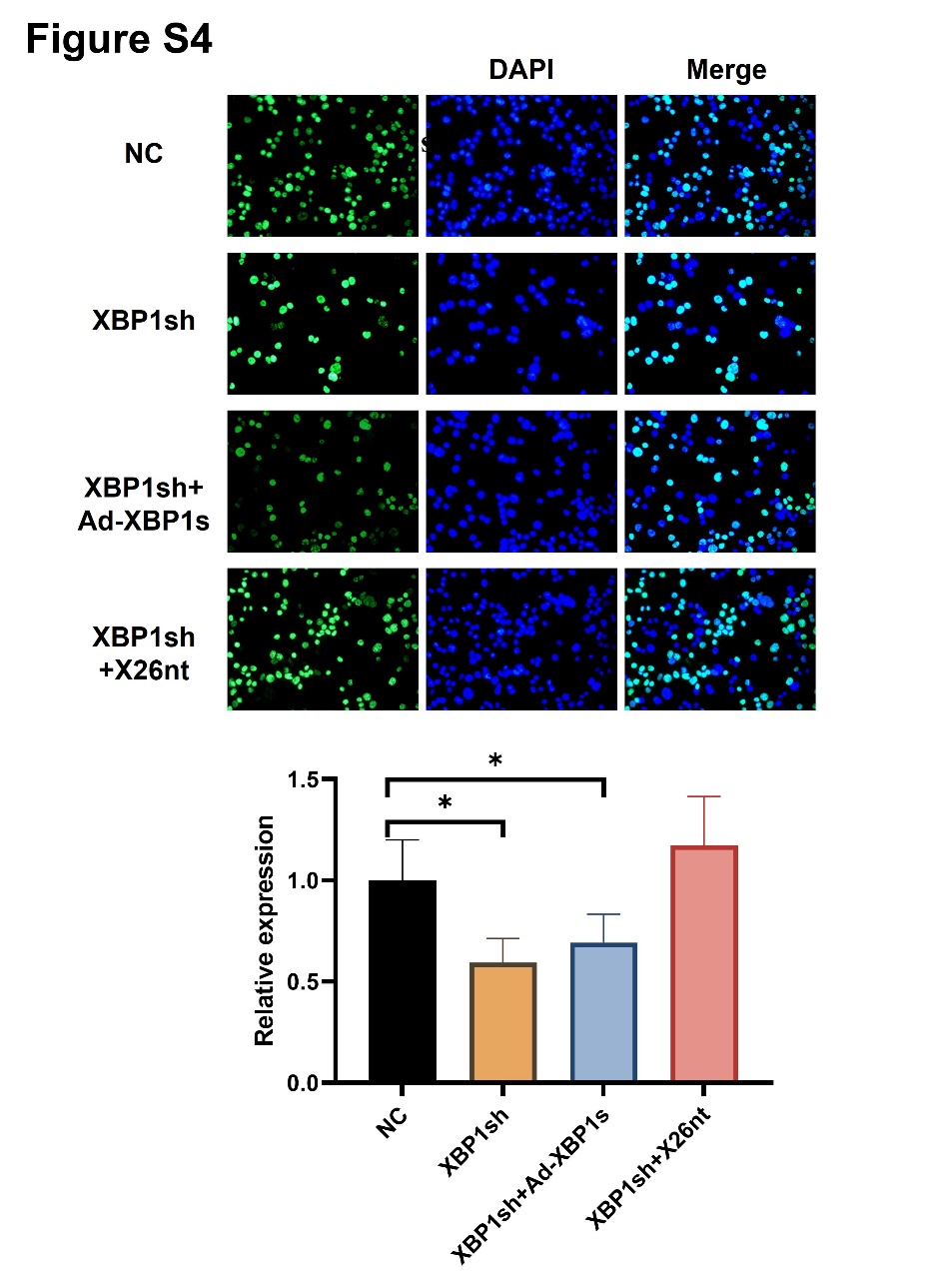


**Figure S4: *X26nt* promoted the proliferation of HUVECs.**

Proliferation of HUVECs measured by Edu assay. HUVECs were transfected directly with *XBP1* inhibitors (*XBP1sh*), and coculture with *XBP1s* and *X26nt* (*n = 3*), **P<0.05*.

**Figure S5**


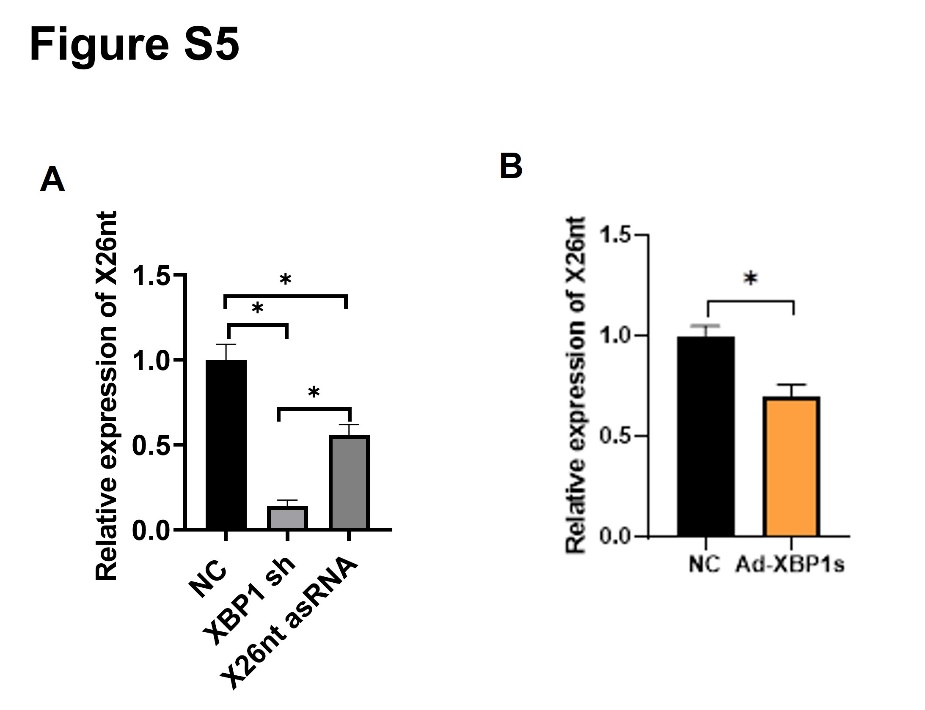


**Figure S5: *X26nt* promoted the expression of *CCND1*.**

(A) Expression of *X26nt* is lower in *XBP1* knocked down cells (*XBP1 sh*) than in *X26nt* knocked down cells (*X26nt asRNA*). (B) *X26nt* is reduced in *XBP1* overexpressed cells (*Ad-XBP1s*) (*n = 3*), **P<0.05*.

**Figure S6**


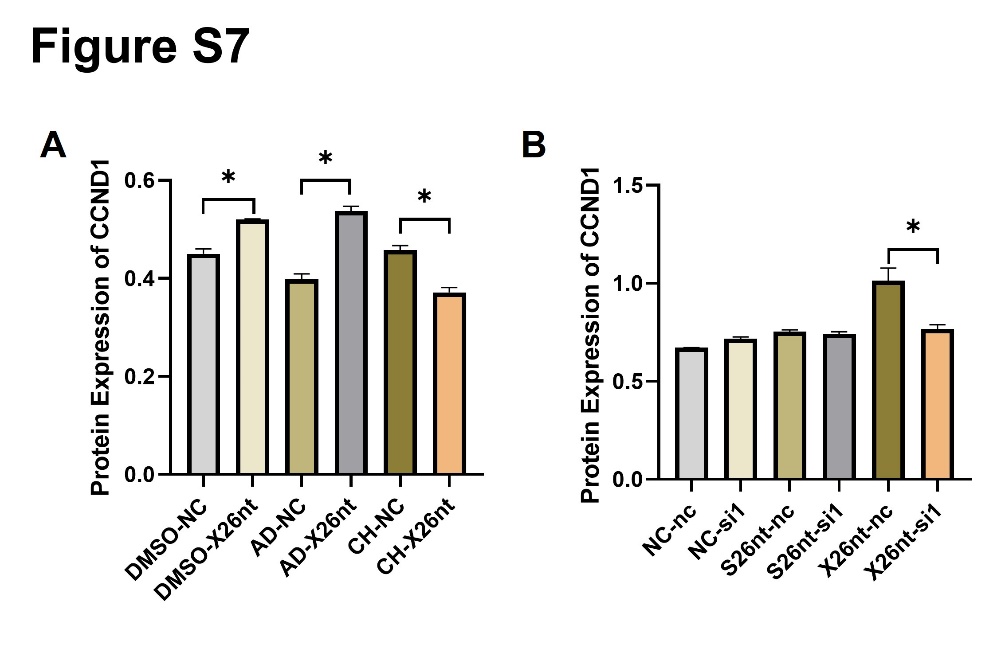


**Figure S6: Western Blot grey scale analysis.**

(A) Western Blot grey scale analysis of Fig 1G. (B) Western Blot grey scale analysis of Fig 1L.

**Figure S7**


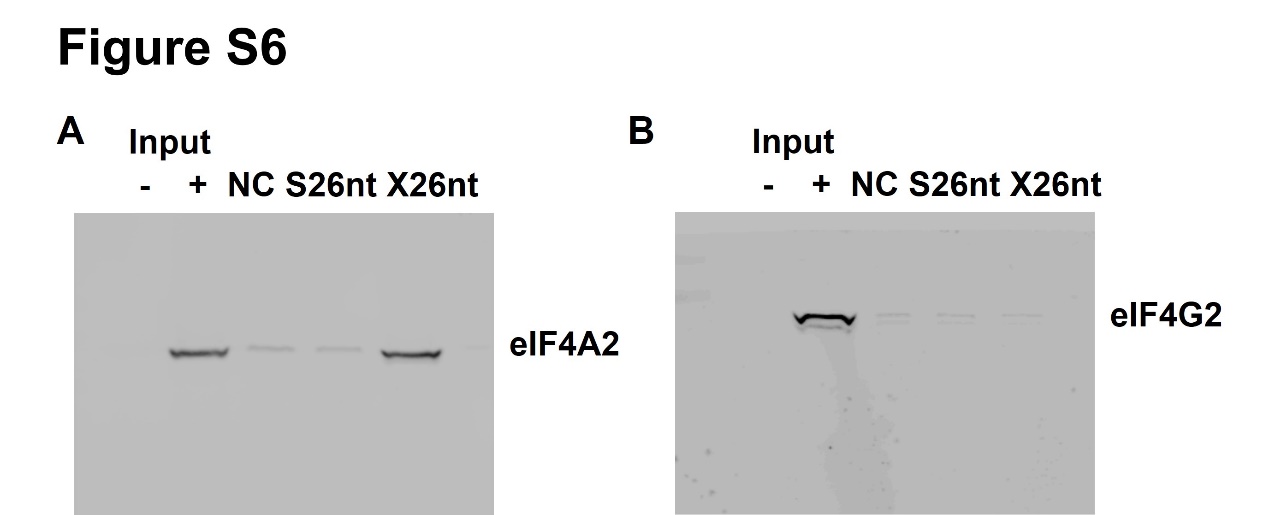


**Figure S7: *X26nt* cannot combine with EIF4G2.**

Western blot of EIF4G2 in cells cocultured with *NC*, *S26nt* and *X26nt*.

**Figure S8**


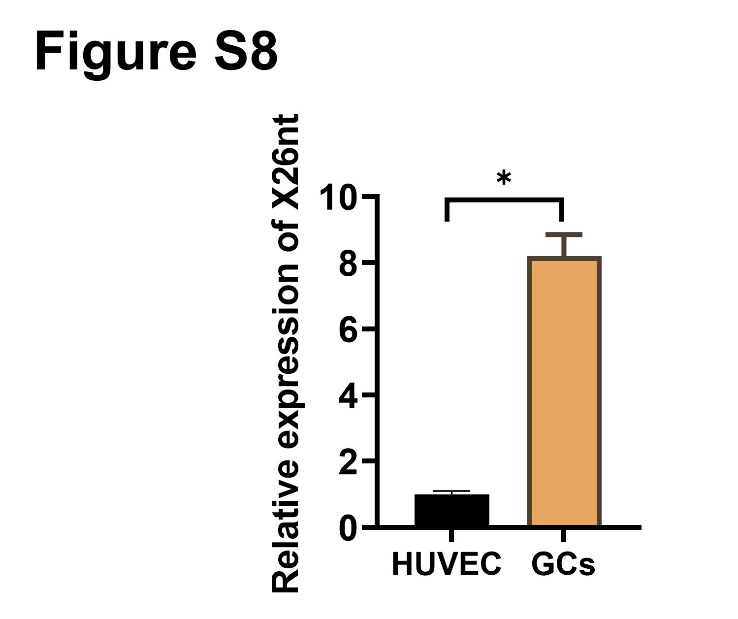


**Figure S8: X26nt is higher in GCs than HUVECs.**

Quantitative analysis of X26nt in HUVEC and GCs.

**Figure S**
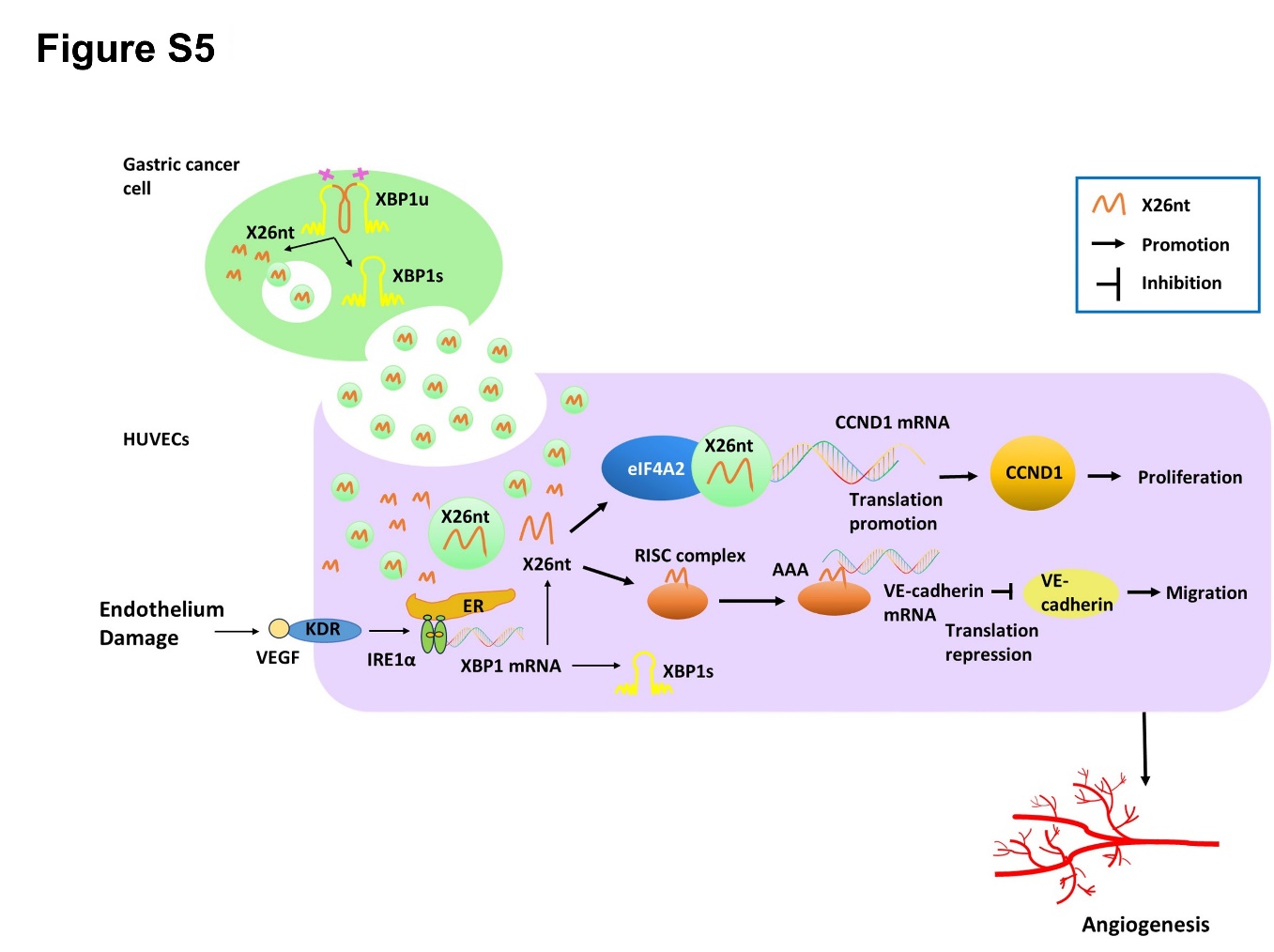
**9**

**Figure S9: Schematic illustration of *X26nt* induced angiogenesis.**

Upon GC cell proliferation, the by-product *X26nt*, which is formed by *XBP1u* splicing, is secreted out through GC cell-derived exosomes. Endothelium damage also facilitates *XBP1* splicing and therefore promoting *X26nt* by activating IRE1α. *X26nt* binds to *CCND1* mRNA 5’UTR, upregulating cell cycle and promoting endothelial cell proliferation. It also combines with VE cadherin mRNA 3′UTR, which reduces the expression of VE-cadherin and promotes endothelial cell migration and tube formation, thereby promoting tumor angiogenesis.
