# Supplementary material for: X26nt-mediated recruitment of eIF4A2 facilitates CCND1 translation to drive endothelial cell cycle progression
Source: Genes Dis. 2025 May 2;12(6):101667. doi: 10.1016/j.gendis.2025.101667 (PMC12281298; doi:10.1016/j.gendis.2025.101667)
Supplement: Multimedia component 2 [file mmc2.docx]

**Figure 1: *X26nt* can increase *CCND1* through binding to the 5’UTR of *CCND1* mRNA mediated by EIF4A2, facilitate proliferation of HUVECs, promote tumor angiogenesis and therefore contribute to tumor progression.**

(A) HUVECs were synchronized at late G1 phase by double thymidine block, then released to re-enter the cell cycle. Cells were harvested at time indicated after release. PCR analysis of XBP1 (total) and XBP1s expression after released in different times (n=3). (B) Quantitative analysis XBP1s/XBP1u rate of (A), (C) HUVECs were infected with Ad-XBP1s and Ad-XBP1u, its expression of XBP1 and CCND1 was analyzed by PCR. Ad-null was introduced as control. (D) Western blot of XBP1s, CCNA1, CCND1, P130, RB, E2F2 of Ad-XBP1s-infected-HUVECs collected at 24h, 48h, 72h. GAPDH was introduced as loading control. (E) Flow cytometry analysis shows the cell cycle distribution of HUVECs transfected with negative control (NC) or XBP1 shRNA, and cells transfected with XBP1 shRNA cocultured with X26nt. Bar graphs showing the percentages of HUVECs in the G0/G1, S and G2/M phases. (F) Western blot analysis of cytosol and nuclear protein collected from HUVECs cocultured with negative control, S26nt or X26nt. TUBULIN and LAMB1 were introduced as loading controls. (G)The protein expression of CCND1 with the treatment of actinomycin (AD) and cycloheximide (CH) in HUVECs cocultured with or without X26nt. (H) Schematic showing potential X26nt binding sites in the CCND1 mRNA 5′UTR. (I) The luciferase assay of HUVECs cells co-transfected firefly luciferase reporter plasmid containing either wild-type (pmirGLO-h. CCND1 WT) or mutant CCND1 mRNA 5′UTR with pmirGLO-h. CCND1 M, cocultured with X26nt or NC-RNA, *P < 0.05. (J) Western blot of EIF4A2 in cells cocultured with NC, S26nt and X26nt. (K) Immunoblots of EIF4A2 in HUVECs transfected with three different si-EIF4A2 plasmids, the scrambled EIF4A2 inhibitors were transfected as control (nc). (L) Western blot analysis of CCND1 in nc and si-1 HUVECs co-cultured with S26nt and X26nt, β-ACTIN was introduced as loading control. (M-P) The formed tumors from BGCs transfected with X26nt-overexpressing (OE-X26nt) lentivirus and control lentivirus (Control) were isolated and compared. Analysis of tumor diameter, volume and weight in each group is shown. (Q-T) The BGC-823 cells (BGCs) were subcutaneously injected into the BALB/c nude mice to create a tumor implanted model. When the tumor appeared, X26nt antisense RNA (As-X26nt) plasmid and mock control (NC) were injected to the tumor every other day before the mice were sacrificed. Analysis of tumor diameter volume and weight in each group is shown. (U-X) Immunofluorescence staining of Cycling D1(CCND1)/DAPI in tumor treated with X26nt asRNA plasmid and NC, as well as X26nt OE lentivirus and control, *P<0.05 and **P<0.01 versus the control. Columns, mean (B, C, D, E, F, G, I, J, K, L, n=3; M-X, n=4); bars, standard deviations. Data presented are representative or average of three independent experiments.

**Figure S1: XBP1 splicing is a cell cycle-related event.**

(A) HUVECs were synchronized at late G1 phase by double thymidine block, then released to re-enter the cell cycle. Cells were harvested at time indicated after release, followed by cell cycle analysis. Data presented are representative or average of three independent experiments. (B) 3% low melting agarose gel was used to separate XBP1u (252bp) and XBP1s (226bp).

**Figure S2: XBP1 splicing promoted expression of CCND1 mediated by VEGF.**

XBP1 and IRE1α knockdown cells were constructed and treated with VEGF. Western blot analysis showed the expression of CCND1 and E2F2. GAPDH was introduced as loading control (n = 3), *P<0.05.

**Figure S3: XBP1s showed suppressed effect on some cell cycle-related proteins.**

HUVECs were infected with Ad-XBP1s for 24h, 48h, 72h and collected protein. (A)Western blot of CCNB1, CCNE1, E2F2. GAPDH was introduced as loading control. (B) Western blot of pRb and Rb, as well as analysis of Rb/pRb ratio. GAPDH was introduced as loading control (n = 3), *P<0.05.

**Figure S4: X26nt promoted the proliferation of HUVECs.**

Proliferation of HUVECs measured by Edu assay. HUVECs were transfected directly with XBP1 inhibitors (XBP1sh), and coculture with XBP1s and X26nt (n = 3), *P<0.05.

**Figure S5: X26nt promoted the expression of CCND1.**

(A) Expression of X26nt is lower in XBP1 knocked down cells (XBP1 sh) than in X26nt knocked down cells (X26nt asRNA). (B) X26nt is reduced in XBP1 overexpressed cells (Ad-XBP1s) (n = 3), *P<0.05.

**Figure S6: Western Blot grey scale analysis.**

(A) Western Blot grey scale analysis of Fig 1G. (B) Western Blot grey scale analysis of Fig 1L.

**Figure S7: X26nt cannot combine with EIF4G2.**

Western blot of EIF4G2 in cells cocultured with NC, S26nt and X26nt.

**Figure S8: X26nt is higher in GCs than HUVECs.**

Quantitative analysis of X26nt in HUVEC and GCs.

**Figure S9: Schematic illustration of X26nt induced angiogenesis.**

Upon GC cell proliferation, the by-product X26nt, which is formed by XBP1u splicing, is secreted out through GC cell-derived exosomes. Endothelium damage also facilitates XBP1 splicing and therefore promoting X26nt by activating IRE1α. X26nt binds to CCND1 mRNA 5’UTR, upregulating cell cycle and promoting endothelial cell proliferation. It also combines with VE cadherin mRNA 3′UTR, which reduces the expression of VE-cadherin and promotes endothelial cell migration and tube formation, thereby promoting tumor angiogenesis.
